# Activation of σ^28^-dependent transcription in *Escherichia coli* by the cyclic AMP receptor protein requires an unusual promoter organization

**DOI:** 10.1111/j.1365-2958.2009.06913.x

**Published:** 2009-11-04

**Authors:** Kerry Hollands, David J Lee, Georgina S Lloyd, Stephen J W Busby

**Affiliations:** School of Biosciences, University of BirminghamEdgbaston, Birmingham, UK

## Abstract

The *Escherichia coli aer* regulatory region contains a single promoter that is recognized by RNA polymerase containing the flagellar sigma factor, σ^28^. Expression from this promoter is dependent on direct activation by the cyclic AMP receptor protein, which binds to a target centred 49.5 base pairs upstream from the transcript start. Activator-dependent transcription from the *aer* promoter was reconstituted *in vitro*, and a tethered inorganic nuclease was used to find the position of the C-terminal domains of the RNA polymerase α subunits in transcriptionally competent open complexes. We report that the ternary activator–RNA polymerase–*aer* promoter open complex is organized differently from complexes at previously characterized promoters. Among other *E. coli* promoters recognized by RNA polymerase containing σ^28^, only the *trg* promoter is activated directly by the cyclic AMP receptor protein. The organization of the different promoter elements and the activator binding site at the *trg* promoter is the same as at the *aer* promoter, suggesting a common activation mechanism.

## Introduction

The cyclic AMP receptor protein (CRP, also known as the catabolite activator protein, CAP) is a global transcription factor, which plays a central role in the control of metabolism in *Escherichia coli* and other enteric bacteria (Kolb *et al.*, 1993; Barrett *et al.*, 2005). CRP, which is functional as a homodimer, recognizes 22 bp target sequences, with the consensus 5′-AAATGTGATCTAGATCACATTT-3′. At most target promoters studied to date, CRP activates transcription by making one or more direct contacts with RNA polymerase, and there appear to be two major classes of simple CRP-activated promoters (Busby and Ebright, 1999). At Class I promoters, CRP binds upstream of the promoter −35 element, at a site centred at position −61.5 (i.e. between base pairs 61 and 62 upstream from the transcript start), or further upstream, and an activating region (AR1) in the downstream subunit of the CRP dimer makes contact with the C-terminal domain of one of the two RNA polymerase α subunits (αCTD). At Class II promoters, CRP binds at a target that overlaps the promoter −35 element and is usually centred at position −41.5. AR1 in the upstream subunit of the CRP dimer interacts with αCTD, while a second activating region (AR2) in the downstream subunit interacts with the N-terminal domain of one of the two RNA polymerase α subunits (αNTD) (Busby and Ebright, 1999).

Although the mechanisms of activation by CRP at both classes of promoter have been scrutinized in detail, most studies have focused on a small number of natural and synthetic model promoters, so it is unclear whether the findings apply to all target promoters. Genomic approaches have now identified scores of new targets for CRP (Tan *et al.*, 2001; Brown and Callan, 2004; Gosset *et al.*, 2004; Zheng *et al.*, 2004; Grainger *et al.*, 2005). This affords an opportunity to study CRP-dependent regulation at a range of naturally occurring promoters, and to uncover novel mechanisms of regulation by CRP. Previously, Hollands *et al.* (2007) investigated the action of CRP at 11 such uncharacterized targets in the *E. coli* K-12 genome. One of these was located in the regulatory region of the *aer* gene, which encodes an aerotaxis sensor protein that controls movement of bacterial cells in response to the availability of oxygen and other electron acceptors in the environment (Bibikov *et al.*, 1997; Rebbapragada *et al.*, 1997; Taylor *et al.*, 1999). CRP binding upstream of *aer* was first detected by Grainger *et al.* (2005) in a whole genome chromatin immunoprecipitation analysis, and it was subsequently shown that CRP activates transcription by binding to a single DNA site in the *aer* regulatory region (Hollands *et al.*, 2007).

Recent transcriptome analyses have indicated that expression of *aer* in both *E. coli* and *Salmonella enterica* serovar Typhimurium requires an alternative σ factor, σ^28^ (Frye *et al.*, 2006; Zhao *et al.*, 2007). Recall that the RNA polymerase σ subunit is a dissociable promoter specificity factor that binds to core RNA polymerase (E) to form the RNA polymerase holoenzyme (Eσ), which can recognize promoter sequences and initiate transcription (Burgess *et al.*, 1969). Most bacteria contain multiple σ factors that recognize different promoter −10 and −35 elements. A primary σ factor (σ^70^ in *E. coli*) drives the transcription of genes with ‘housekeeping’ functions, while a number of alternative σ factors direct transcription of particular sets of genes in response to environmental signals or stresses, or function to control development (Ishihama, 2000; Gruber and Gross, 2003). σ^28^, which is encoded by the *fliA* gene, is the most widely distributed alternative σ factor (Koo *et al.*, 2009; Smith and Hoover, 2009), and controls the transcription of operons required for flagellar filament assembly and for the regulation of motility and chemotaxis in a large number of Gram-positive and Gram-negative bacteria (Chilcott and Hughes, 2000).

Most studies on transcription activation by CRP have been concerned with promoters recognized by RNA holoenzyme containing σ^70^ (Eσ^70^). Here, we report the first investigation into the direct regulation by CRP of transcription by RNA polymerase containing σ^28^ (Eσ^28^). We show that *aer* is transcribed from a single σ^28^-dependent promoter that is activated by CRP binding at a location different from any previously characterized CRP-activated promoter. We also show that CRP directly activates transcription from a second σ^28^-dependent promoter that has a similar organization.

## Results and discussion

### Transcription from the *aer* regulatory region requires both CRP and σ^28^*in vivo*

**Fig. 1:**
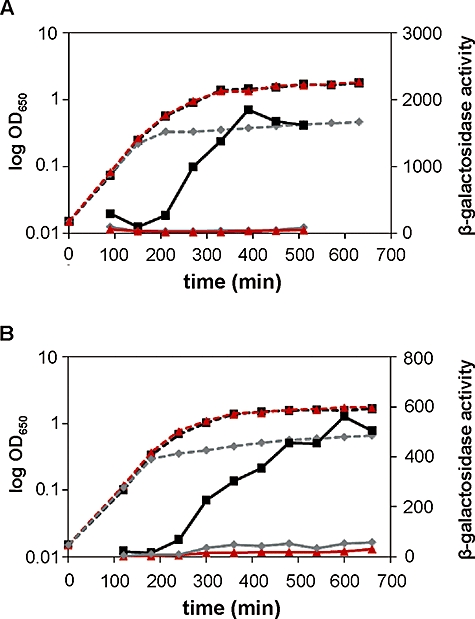
CRP and σ^28^ dependency of *aer* promoter activity throughout growth. A. Requirement for CRP and σ^28^ in a strain expressing σ^28^ from the chromosomal *fliA* promoter. The figure shows OD_650_ values (dashed lines) and β-galactosidase activities (in Miller units; solid lines) measured throughout growth in *Escherichia coli* K-12 strain M182 (black lines), M182 Δ*crp* (grey lines) or M182 Δ*fliA* (red lines), each carrying the aer200::*lacZ* fusion cloned in pRW50. B. Requirement for CRP and σ^28^ in a strain expressing σ^28^ from a CRP-independent promoter on plasmid pKXH100. The figure shows OD_650_ values (dashed lines) and β-galactosidase activities (in Miller units; solid lines) measured in strain M182 Δ*fliA* containing pKXH100 (CRP^+^ FliA^+^ black lines), strain M182 Δ*fliA*Δ*crp* containing pKXH100 (CRP^–^ FliA^+^ grey lines) or strain M182 Δ*fliA* containing ‘empty’ pET21a (CRP^+^ FliA^–^; red lines), each carrying the aer200::*lacZ* fusion cloned in pRW50.

To study the effects of CRP and σ^28^ on expression of *aer*, we cloned a DNA fragment covering the *aer* gene regulatory region (aer200; Hollands *et al.*, 2007) into a low-copy-number *lac* expression vector, pRW50, and we measured the activity of the resulting aer200::*lacZ* fusion in *E. coli* K-12 Δ*lac* strain M182 and derivatives containing deletions of either the *crp* or *fliA* gene. Results presented in Fig. 1A (black lines) show that, in M182, there is a large increase in promoter activity during late exponential phase that decreases on entry into stationary phase. This is consistent with the findings of Barembruch and Hengge (2007), who observed a similar pattern of expression for the σ^28^-dependent *flgM* promoter, and correlates with an accumulation of σ^28^ protein during late exponential phase followed by a decline in σ^28^ levels once the culture enters stationary phase (K. Hollands, unpubl. data; Barembruch and Hengge, 2007). In the Δ*fliA* and Δ*crp* backgrounds (Fig. 1A, red and grey lines), promoter activity remained at a basal level throughout the growth cycle. This confirms that both CRP and σ^28^ are essential for expression from the *aer* regulatory region *in vivo*. However, this experiment is complicated by the fact that σ^28^ expression is dependent on CRP. This is because CRP is required to activate transcription of the *flhDC* operon that encodes an essential activator of transcription from the *fliA* promoter (Soutourina *et al.*, 1999). Indeed, Western blot analysis confirms that no σ^28^ protein is present in strain M182 Δ*crp* (Fig. S1, lanes 1–3).

To investigate the action of CRP at the *aer* regulatory region, independent of the indirect effect of CRP on σ^28^ levels, we established an experimental system in which expression of σ^28^ is uncoupled from CRP. To do this, we used Δ*fliA* derivatives of M182 and M182 Δ*crp* that had been transformed with pKXH100, which encodes *fliA* under the control of a CRP-independent promoter. Western blot analysis confirms that, in this system, expression of σ^28^ does not require CRP (Fig. S1, lanes 4–6). We measured expression of the aer200::*lacZ* fusion in pRW50 in these strains. Data illustrated in Fig. 1B show that, in M182 Δ*fliA* pKXH100, the activity of the aer200::*lacZ* fusion follows a similar pattern to that in strain M182 (which expresses *fliA* from the chromosome), except that the increase in promoter activity occurs later in growth, once the culture begins to enter stationary phase. This correlates with a delayed increase in σ^28^ protein levels in this background (K. Hollands, unpubl. results). In the absence of CRP, promoter activity remains low throughout the growth cycle, showing that the requirement for CRP for expression from the *aer* regulatory region is independent of the effect of CRP on σ^28^ levels. We conclude that CRP must function directly at the *aer* regulatory region, and this is consistent with the previous observation that introducing mutations into the DNA target for CRP upstream of *aer* prevents CRP-dependent activation of the aer200::*lacZ* fusion (Hollands *et al.*, 2007).

### *aer* is transcribed from a single σ^28^-dependent promoter *in vivo*

**Fig. 2:**
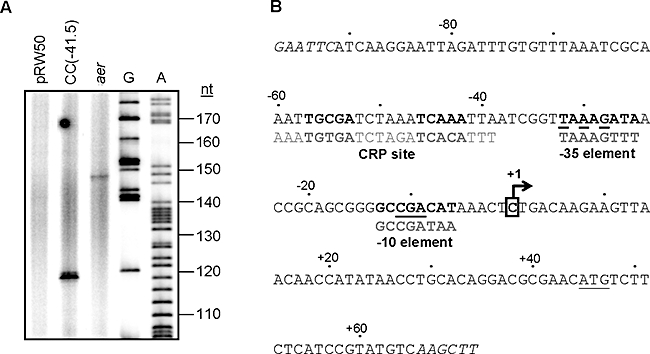
Identification of the *aer* transcript start site. A. The figure shows the result of primer extension analysis using RNA extracted from strain M182, carrying the aer200 fragment cloned in pRW50, grown aerobically to mid-exponential phase (OD_650_ 0.4–0.6) in LB medium. Control primer extension reactions were also carried out using RNA extracted from M182 cells containing ‘empty’ pRW50, or pRW50 carrying the CC(−41.5) promoter, whose transcript start site is known, and which gives a primer extension product of 118 nt in pRW50. The sizes of primer extension products were determined by calibration against sequencing reactions (lanes G and A). B. The figure shows the base sequence of the non-template strand of the aer200 promoter fragment used in this work. The transcript start site proposed here is boxed, and the *aer* translation start codon is underlined. The proposed −10 and −35 octamer elements of the σ^28^-dependent *aer* promoter, and the DNA site for CRP, are highlighted in bold. The consensus sequence for each DNA element is indicated in grey below the sequence (Busby and Ebright, 1999; Koo *et al.*, 2009). Bases in the −10 and −35 elements that were targeted for mutational analysis are underlined in bold. The EcoRI and HindIII sites flanking the aer200 fragment are shown in italics, and the sequence is numbered with the *aer* transcript start site as +1.

Although the *aer* regulatory region has been predicted as a target for Eσ^28^ (Park *et al.*, 2001; Frye *et al.*, 2006; Zhao *et al.*, 2007), the promoter determinants required for transcription initiation have not been identified experimentally. To define the DNA elements required for σ^28^-dependent transcription of *aer*, we began by mapping the *aer* transcript start site by primer extension analysis, using the aer200 promoter fragment cloned in pRW50. This yielded a single extension product approximately 148 nucleotides in length (Fig. 2A), which places the transcript start point at the position labelled +1 in Fig. 2B. This falls 6 bp downstream from the −10 octamer element for a σ^28^-dependent promoter predicted by Park *et al.* (2001). To examine the importance of this promoter, we constructed derivatives of the aer200 fragment containing point mutations in the putative −10 and −35 elements (Table 1 and Fig. 2B). In the −10 element, we targeted the highly conserved 5′-CGA-3′ motif, from positions −11 to −9, because mutations in this motif result in a loss of σ^28^-dependent transcription from other σ^28^-dependent promoters, both *in vivo* and *in vitro* (Yu *et al.*, 2006; Wozniak and Hughes, 2008). In the −35 octamer, we targeted positions −32T and −30A, which are also highly conserved and are important for σ^28^-dependent transcription from the *Salmonella flgM* promoter, and position −28, which has more minor effects on *flgM* promoter activity (Wozniak and Hughes, 2008). Each mutant promoter fragment was cloned into pRW50, and expression of the resulting promoter::*lacZ* fusions was measured in the CRP^+^ FliA^+^, CRP^–^ FliA^+^ and CRP^+^ FliA^–^ backgrounds. Results listed in Table 1 show that the substitutions in the −10 element had the greatest effect on promoter activity, reducing expression from the *aer* regulatory region to the level observed in the absence of σ^28^. Mutations at positions −32, −30 and −28 in the −35 element also severely reduced promoter activity. We conclude that the proposed −10 and −35 elements are essential for σ^28^-dependent transcription of *aer*, and, together with the transcript start site data, this argues that *aer* is expressed from a single promoter, at least under the conditions tested here.

**Table 1:** Effect of mutations in the −10 and −35 elements on *aer* promoter activity.

		β-Galactosidase activity		
Promoter fragment	Promoter sequence	CRP^+^ FliA^+^	CRP^–^ FliA^+^	CRP^+^ FliA^–^
aer200	TAAAGATA-n_11_-GCCGACAT	223 ± 27	53 ± 6	12 ± 1
aer206	TAAAGATA-n_11_-GCGCTCAT	12 ± 1	32 ± 1	11 ± 1
aer213	CAAAGATA-n_11_-GCCGACAT	23 ± 2	31 ± 1	23 ± 1
aer214	TATAGATA-n_11_-GCCGACAT	26 ± 1	43 ± 1	14 ± 1
aer224	TAAAAATA-n_11_-GCCGACAT	50 ± 2	36 ± 1	12 ± 1
Consensus:	TAAAGTTT-n_11_-GCCGATAA			

The table lists β-galactosidase activities (in Miller units) measured in strain M182 Δ*fliA* containing pKXH100 (CRP^+^ FliA^+^), strain M182 Δ*fliA*Δ*crp* containing pKXH100 (CRP^–^ FliA^+^) or strain M182 Δ*fliA* containing ‘empty’ pET21a (CRP^+^ FliA^–^), each carrying different *aer* promoter::*lacZ* fusions cloned in pRW50 and grown to late exponential phase (OD_650_ 0.9–1.1) in LB medium. The aer200 fragment carries the wild-type *aer* promoter, the aer206 fragment carries three point mutations in the proposed −10 element, and the aer213, aer214 and aer224 fragments carry single point mutations in the proposed −35 octamer. The sequence of the −10 and −35 elements of the σ^28^-dependent *aer* promoter is listed for each fragment, and the location of base changes in each of the mutant promoter derivatives is underlined. The consensus sequence for a σ^28^-dependent promoter is shown below the table. Data listed are averages from at least three independent experiments, shown ± one standard deviation.

### Transcription initiation at the *aer* promoter *in vitro*

**Fig. 3:**
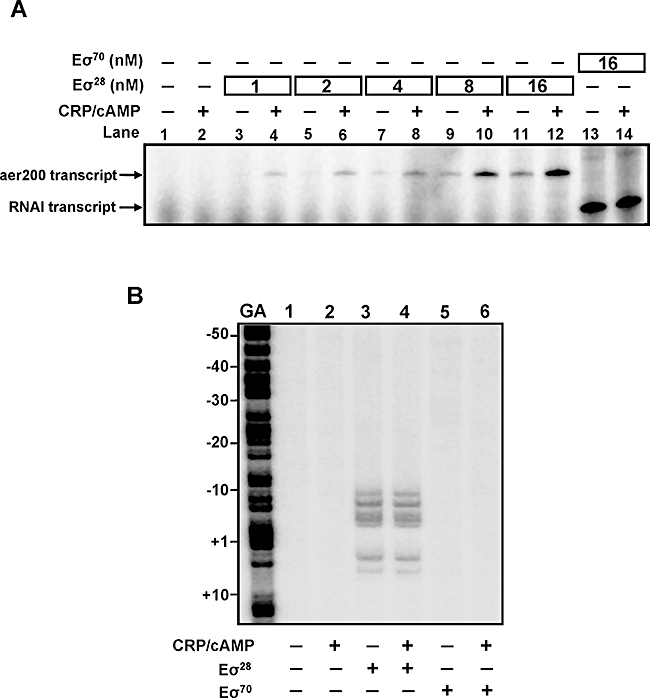
Sigma factor selectivity and effect of CRP at the aer promoter *in vitro*. A. *In vitro* transcription from the *aer* promoter. The figure shows the transcripts produced in multi-round *in vitro* transcription assays using the aer200 fragment cloned in pSR, incubated with various concentrations of Eσ^28^ or Eσ^70^, and in the presence or absence of 100 nM CRP and 0.2 mM cAMP, as indicated. The locations of the σ^28^-dependent *aer* transcript and the σ^70^-dependent RNAI control transcript are indicated. B. Open complex formation at the *aer* promoter. The figure shows the results of KMnO_4_ footprinting using an aer200 PstI-HindIII fragment, end-labelled on the template strand and incubated with a final concentration of 50 nM Eσ^28^ or Eσ^70^, in the presence or absence of 100 nM CRP and 0.2 mM cAMP, as indicated. The gel was calibrated using a Maxam-Gilbert ‘G + A’ sequencing reaction, and is numbered with respect to the *aer* transcript start site.

Next, we sought to confirm our *in vivo* findings by examining the σ factor selectivity and CRP dependence of the *aer* promoter *in vitro*. We began by cloning the aer200 fragment upstream of the λ*oop* terminator in plasmid pSR, and tested the ability of purified Eσ^28^ and Eσ^70^ to drive transcription from the *aer* promoter in an *in vitro* multi-round transcription assay, in the presence and absence of purified CRP and cAMP (Fig. 3A). In this system, transcription initiating at the *aer* promoter terminates at the λoop terminator to generate a 158-base transcript that can be identified by electrophoresis. In the presence of Eσ^28^, a single transcript was observed (Fig. 3A, lanes 3–12). At low Eσ^28^ concentrations, this transcript is detected only in the presence of CRP (lanes 3–6), although some transcript is generated in the absence of CRP as the RNA polymerase concentration is increased (lanes 7–12). At even higher concentrations of Eσ^28^, transcription becomes completely independent of CRP (data not shown). The *aer* transcript generated by Eσ^28^ is not detected in reactions using Eσ^70^ (Fig. 3A, lanes 13 and 14). Instead, a single CRP-independent transcript is produced, which corresponds to the 108-base RNAI control transcript that originates from the pSR replication origin.

To confirm that *in vitro* transcription initiates from the same promoter defined in our *in vivo* experiments, promoter unwinding by RNA polymerase was monitored by using KMnO_4_ to probe for single-stranded regions of DNA (Fig. 3B). In the presence of Eσ^28^ (lanes 3 and 4), KMnO_4_-reactive bands appeared from positions −10 to +4, indicative of promoter melting around the −10 element of the σ^28^-dependent promoter highlighted in Fig. 2B. This was observed both in the presence and in the absence of CRP, which is consistent with our finding that transcription initiation by Eσ^28^ is independent of CRP *in vitro* at the high RNA polymerase concentrations used in these reactions. Incubation with Eσ^70^ did not result in promoter melting around the *aer* transcript start site, either in the presence or in the absence of CRP (Fig. 3B, lanes 5 and 6). Taken together, the *in vitro* data confirm that *aer* is transcribed from a single, σ^28^-dependent promoter that is activated by CRP when the RNA polymerase concentration is limited. The observation that the *aer* promoter becomes less dependent on CRP at higher RNA polymerase concentrations suggests that CRP activates transcription by recruitment of RNA polymerase (Rhodius *et al.*, 1997).

### Transcription activation at the *aer* promoter requires CRP binding at an atypical location

**Fig. 4:**
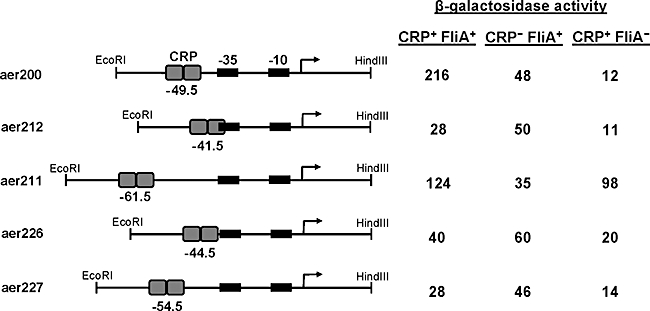
Effect of moving the DNA site for CRP on activation of the *aer* promoter. The figure shows schematic diagrams of the wild-type aer200 promoter fragment, and derivatives in which the DNA site for CRP has been moved to position −41.5 (aer212), −61.5 (aer211), −44.5 (aer226) or −54.5 (aer227). The transcript start sites are indicated by arrows, the locations of the promoter −10 and −35 elements are indicated by black rectangles and the DNA site for CRP is shaded grey. The figure also shows the β-galactosidase activities (in Miller units) measured in strain M182 Δ*fliA* containing pKXH100 (CRP^+^ FliA^+^), strain M182 Δ*fliA*Δ*crp* containing pKXH100 (CRP^–^ FliA^+^) or strain M182 Δ*fliA* containing ‘empty’ pET21a (CRP^+^ FliA^–^), each carrying the different *aer* promoter::*lacZ* fusions cloned in pRW50. Cells were grown to late exponential phase (OD_650_ 0.9–1.1) in LB medium. Data shown are averages from three independent experiments, with a standard deviation of less than 10%.

Mutational analysis showed that CRP-dependent activation of the aer200::*lacZ* fusion requires CRP binding to the single DNA target indicated in Fig. 2B (Hollands *et al.*, 2007). This target site is centred 49.5 bp upstream from the transcript start site, which falls between the typical Class I location of position −61.5 and the Class II location of position −41.5. To investigate whether CRP can activate σ^28^-dependent transcription from positions −41.5 or −61.5 at the *aer* promoter, we constructed a deletion or insertion in the aer200 fragment to make the aer212 and aer211 fragments (Fig. 4, upper three panels). These fragments were cloned into pRW50, and expression of the resulting promoter::*lacZ* fusions was measured in the CRP^+^ FliA^+^, CRP^–^ FliA^+^ and CRP^+^ FliA^–^ backgrounds. The results illustrated in Fig. 4 show that, when the DNA site for CRP is moved to position −41.5, *aer* promoter activity in the CRP^+^ FliA^+^ strain is reduced to a similar level to that observed in the absence of CRP or σ^28^. This indicates that CRP cannot activate transcription from the *aer* promoter when bound at a Class II location. Moving the CRP site to position −61.5 (aer211) results in a twofold decrease in promoter activity, but, while the residual promoter activity is dependent on CRP, it is independent of σ^28^. The most likely explanation for this is that, here, CRP is activating transcription from an alternative cryptic σ^28^-independent promoter. For example, in the aer211 fragment, a 6 bp sequence, 5′-TAAAGA-3′, is located 32 bp downstream of the DNA site for CRP and this may well generate a weak Class II CRP-dependent promoter served by Eσ^70^ (recall that the consensus −10 hexamer for Eσ^70^ is 5′-TATAAT-3′).

Next, we used the same system to monitor the effects of making a 5 bp deletion or insertion between the DNA site for CRP and the −35 element at the *aer* promoter. Both the deletion, which moved the DNA site for CRP to position −44.5 (aer226), and the insertion, which moved the DNA site for CRP to position −54.5 (aer227), resulted in a reduction in promoter activity in the CRP^+^ FliA^+^ strain to the basal level observed in the absence of CRP (Fig. 4, lower two panels). This indicates that CRP is unable to activate transcription from the *aer* promoter when its DNA site is moved to the opposite face of the DNA helix. These experiments argue that optimal activation of σ^28^-dependent transcription requires CRP binding at position −49.5.

### Location of RNA polymerase α C-terminal domains at the *aer* promoter

Activation by CRP at both Class I and Class II σ^70^-dependent promoters requires a contact between CRP activating region 1 (AR1) and αCTD. Previous work showed that CRP-dependent activation at the *aer* promoter also requires AR1 (Hollands *et al.*, 2007), which likely functions by contacting αCTD in Eσ^28^. Because the organization of the *aer* promoter is unlike that at Class I or Class II CRP-dependent promoters, it is unclear whether the interaction between AR1 and αCTD occurs via the upstream or downstream subunit of dimeric CRP bound at the promoter. To address this, we mapped the location of αCTD binding at the *aer* promoter using purified RNA polymerase that had been labelled with the chemical nuclease reagent iron [S]-1-[*p*-bromoacetamidobenzyl] ethylenediaminetetraacetate (FeBABE) on a single cysteine residue at position 302 in the αCTDs (see *Experimental procedures*). Transcriptionally competent open complexes were formed using the end-labelled aer200 promoter fragment, purified CRP and FeBABE-tagged Eσ^28^, and DNA cleavage by FeBABE was triggered. Analysis of the pattern of DNA cleavage by gel electrophoresis reveals the location of the αCTDs at the *aer* promoter. Note that, in this assay, in most cases, a single Fe-BABE-labelled αCTD will give rise to cleavages in two adjacent minor grooves, as a wave of hydroxyl radicals generated from the Fe-BABE impinges on the target DNA (Lee *et al.*, 2003).

**Fig. 5:**
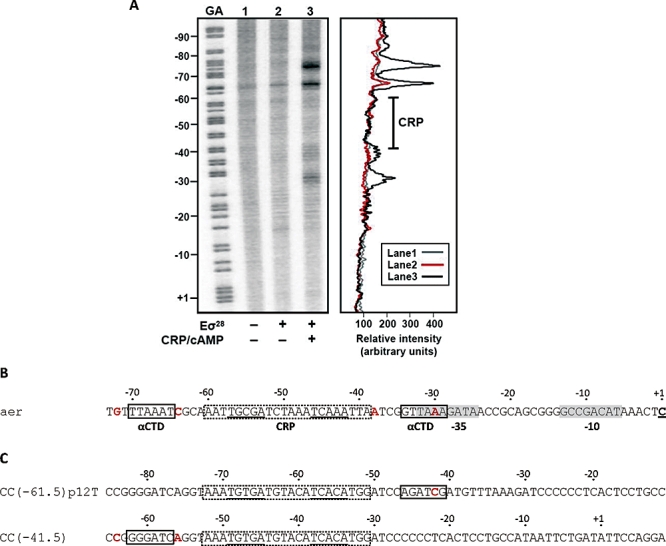
Mapping the location of the RNA polymerase α C-terminal domains at the *aer* promoter. A. The figure shows the results of FeBABE footprinting using the aer200 promoter fragment, and Eσ^28^ tagged with FeBABE at position 302 of the α C-terminal domain. The PstI-HindIII promoter fragment, end-labelled on the template strand, was incubated in the presence or absence of 200 nM FeBABE-tagged Eσ^28^ and 100 nM CRP/0.2 mM cAMP, as indicated. The left hand panel shows an autoradiograph of the 6% polyacrylamide sequencing gel on which the reactions were run. The gel was calibrated using a Maxam-Gilbert ‘G + A’ sequencing reaction, and is numbered with respect to the *aer* transcript start site. The right hand panel shows a plot of the relative intensity of bands down each lane of the gel, with the position of the DNA site for CRP indicated. B. Sequence of the *aer* promoter region, showing the proposed locations of αCTD binding. The −10 and −35 elements of the σ^28^-dependent promoter are shaded grey, the DNA site for CRP is denoted by the dashed box, and the locations of FeBABE-induced DNA cleavage are highlighted in red. The sites where the αCTDs are proposed to contact the DNA (6 bp sequences centred 18–19 bp from the centre of the DNA site for CRP (Benoff *et al.*, 2002)) are indicated by the solid boxes. The sequence is numbered with respect to the *aer* transcript start site. C. Proposed locations of αCTD binding at the model Class I and Class II CRP-dependent promoters described by Lee *et al.* (2003). At each promoter, the DNA site for CRP is denoted by a dashed box, and the locations of FeBABE-induced DNA cleavage are highlighted in red. The sites where αCTD is proposed to contact the DNA are indicated by the solid boxes.

Results presented in Fig. 5A show that, in the presence of CRP and Eσ^28^ (lane 3), DNA cleavage on the template strand of the *aer* promoter is enhanced around positions −72 and −64 upstream of the DNA site for CRP, and around positions −38 and −30 downstream of the CRP site. This indicates that the αCTDs can contact the DNA both upstream and downstream of the bound CRP dimer. In the presence of Eσ^28^, but in the absence of CRP (lane 2), the pattern of DNA cleavage is similar to the background detected in the absence of any protein (lane 1). This suggests that the two αCTDs are positioned at their targets on the DNA only in the presence of CRP. Interestingly, the spacing between the centre of the DNA site for CRP and the downstream FeBABE-induced DNA cleavage at the *aer* promoter is identical to that observed by Lee *et al.* (2003) at a Class I CRP-dependent promoter served by Eσ^70^ (Fig. 5B and C). Similarly, the spacing between the centre of the DNA site for CRP and the upstream FeBABE-induced DNA cleavage is identical to that seen at a Class II CRP-dependent promoter served by Eσ^70^ (Lee *et al.*, 2003). Thus, the juxtaposition between the downstream-bound αCTD and CRP in open complexes at the *aer* promoter appears to be identical to the AR1-mediated juxtaposition between downstream-bound αCTD and CRP at a Class I CRP-dependent promoter. Similarly, the juxtaposition between the upstream-bound αCTD and CRP at the *aer* promoter appears to be identical to the AR1-mediated juxtaposition between upstream-bound αCTD and CRP at a Class II CRP-dependent promoter (Fig. 5B and C).

In the crystal structure of the CRP-αCTD-DNA complex, αCTD contacts approximately 6 bp of DNA spanning a minor groove, centred 18–19 bp from the centre of the DNA site for CRP (Benoff *et al.*, 2002). The locations of the specific DNA cleavages at the *aer* promoter are consistent with binding of the αCTDs at sites centred 18.5 bp both upstream and downstream of the DNA site for CRP (Fig. 5B). These sequences are also AT-rich, a feature associated with DNA binding by αCTD (Gourse *et al.*, 2000).

### Regulation by CRP at another σ^28^-dependent promoter

**Fig. 6:**
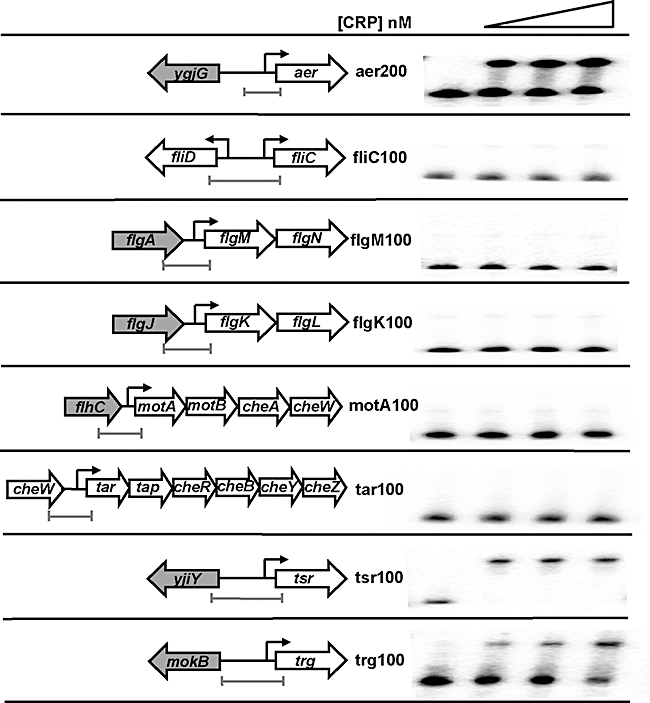
Binding of CRP to σ^28^-dependent promoters *in vitro.* The left hand panels show schematic diagrams of the regulatory regions of the eight well characterized σ^28^-dependent promoters from *E. coli* K-12 (not to scale). σ^28^-dependent genes are indicated by open arrows, while genes that are not known to be σ^28^-dependent are shown as grey arrows. Black lines denote intergenic regions, and black arrows show the locations of known or putative σ^28^-dependent promoters. Grey bars indicate the extent of the EcoRI-HindIII promoter fragments used in this work. The right hand panels show the results of electromobility shift assays using the end-labelled promoter fragments, incubated with 0, 50, 100 or 200 nM CRP, in the presence of 0.2 mM cAMP.

To investigate whether CRP directly regulates Eσ^28^-dependent transcription at other promoters, we used electromobility shift assays to compare the binding of CRP to end-labelled DNA fragments covering the regulatory regions of *aer* and the seven other σ^28^-dependent operons from *E. coli* K-12 strain MG1655 described by Zhao *et al.* (2007). The results, illustrated in Fig. 6, show that CRP binds to a single site in the aer200 fragment, but binding of CRP to specific targets in the *tsr* and *trg* regulatory regions was also detected. Note that bioinformatic analyses had predicted DNA sites for CRP upstream of both *tsr* and *trg* (Robison *et al.*, 1998). The *trg* promoter fragment binds CRP with similar affinity to the *aer* fragment, while CRP binding to the *tsr* fragment is much tighter. No clear binding of CRP was found with the *fliC/fliD*, *flgMN*, *flgKL, motAB/cheAW* or *tar/tap/cheRBYZ* fragments.

**Fig. 7:**
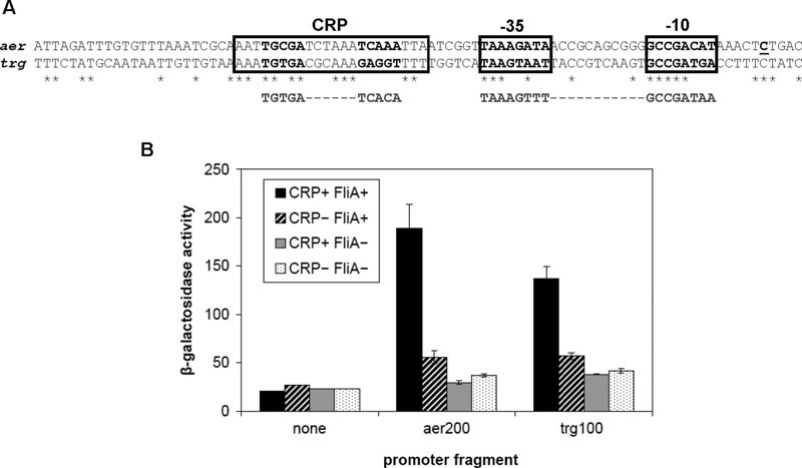
Activation of the σ^28^-dependent *trg* promoter by CRP. A. Sequence alignment of the *aer* and *trg* regulatory regions. The −10 and −35 elements of the σ^28^-dependent *aer* and *trg* promoters, and the DNA sites for CRP, are highlighted. The consensus sequences for CRP and Eσ^28^ binding (Busby and Ebright, 1999; Koo *et al.*, 2009) are shown below the alignment. Asterisks below the sequence specify bases that are identical in the two sequences. B. Effect of CRP on expression from the *trg* promoter. The figure shows β-galactosidase activities (in Miller units) measured in strain M182 Δ*fliA* containing pKXH100 (CRP^+^ FliA^+^), strain M182 Δ*fliA*Δ*crp* containing pKXH100 (CRP^–^ FliA^+^), strain M182 Δ*fliA* containing ‘empty’ pET21a (CRP^+^ FliA^–^) or strain M182 Δ*fliA*Δ*crp* containing ‘empty’ pET21a (CRP^–^ FliA^–^), each carrying the trg100::*lacZ* fusion cloned in pRW50. pRW50 carrying the pUC9 linker was included as a negative control (‘none’), and the aer200::*lacZ* fusion, cloned in pRW50, was included as a positive control. Data shown are averages from three independent experiments, and error bars indicate one standard deviation.

The action of CRP at the *tsr* and *trg* regulatory regions was studied further. In the *tsr* regulatory region, the predicted CRP site is located 132.5 bp upstream of the σ^28^-dependent *tsr* promoter, so it is unlikely that CRP makes direct contact with bound Eσ^28^. Indeed, no direct effect of CRP on gene expression from the *tsr* regulatory region could be detected (K. Hollands, unpubl. data). In contrast, alignment of the DNA sequences of the *trg* and *aer* regulatory regions revealed that the spacing between the predicted DNA sites for CRP and the different elements of the two σ^28^-dependent promoters is identical (Fig. 7A).

To measure the effect of CRP on expression from the *trg* regulatory region, the trg100 promoter fragment was cloned into pRW50, and the activity of each promoter::*lacZ* fusion was measured in the CRP^+^ FliA^+^, CRP^–^ FliA^+^ and CRP^+^ FliA^–^ backgrounds. Recall that, in the conditions used in our experiments, direct effects of CRP on transcription of promoter::*lacZ* fusions in pRW50 can be measured independent of the effect of CRP on σ^28^ levels. Results illustrated in Fig. 7B indicate that expression from the *trg* regulatory region, like the *aer* promoter, is dependent on σ^28^ and is activated by CRP. The conservation of the spacing between the DNA site for CRP and the −10 and −35 elements at the *aer* and *trg* promoters suggests that the mechanisms of transcription activation at the two promoters are similar. Interestingly, the *trg* and *aer* genes encode homologous proteins with similar functions. While Aer is an energy sensor that controls responses to redox signals, Trg is a chemosensor that responds to the monosaccharides ribose and galactose (Taylor *et al.*, 1999).

## Conclusions

Here we have described the first examples of direct activation by CRP of promoters served by RNA polymerase holoenzyme containing the flagellar sigma factor, σ^28^. We showed that transcription of the *E. coli* K-12 *aer* gene is driven by a single σ^28^-dependent promoter, which is activated by CRP binding to a single site positioned 49.5 base pairs upstream of the transcript start site. This location appears optimal for activation. This is in contrast to the situation at previously studied Class I and Class II CRP-dependent promoters where the optimal locations for activation by CRP are positions −61.5 and −41.5 respectively, and where CRP activates only very weakly when bound to a site centred near position −50 (Gaston *et al.*, 1990). Our results argue that the spacing requirements for CRP-dependent activation at promoters served by Eσ^28^ differ from those at promoters served by Eσ^70^. It is possible that promoters recognized by some other alternative σ factors also require CRP binding at unusual locations. For example, at the σ^38^-dependent *csiD* promoter, CRP activates optimally from a DNA site centred at position −68.5 (Germer *et al.*, 2001). From this position, or a site located one helical turn upstream, CRP can activate σ^38^-, but not σ^70^-dependent transcription.

**Fig. 8:**
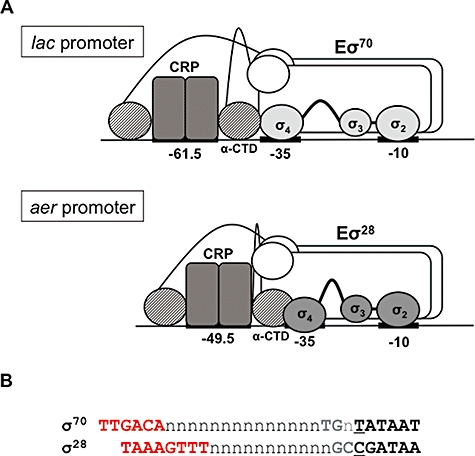
Organization of the CRP-RNA polymerase-DNA complex at the *aer* promoter. A. Comparison of the CRP-RNA polymerase-DNA complexes at σ^70^- and σ^28^-dependent promoters. The upper panel shows a schematic diagram of the CRP-Eσ^70^-DNA ternary complex at the Class I CRP-activated *lac* promoter, where CRP binds at a site centred 61.5 bp upstream of the transcript start site. The lower panel shows a model for the organization of the CRP-Eσ^28^-DNA ternary complex at the *aer* promoter, where CRP binds at a site centred 49.5 bp upstream from the transcript start site. Domain 4 of σ^28^ is proposed to contact the DNA further downstream at the *aer* promoter than does domain 4 of σ^70^ at the *lac* promoter, and this relieves a potential clash with downstream-bound αCTD. B. Spacing between the −10 and −35 elements at σ^70^- and σ^28^-dependent promoters. The figure shows the σ^70^ and σ^28^ promoter consensus sequences, aligned by the position of the upstream edge of the core −10 element (underlined). The core −10 element is shown in black, the extended −10 element in grey and the −35 element in red. Each base in the spacer region is represented by an ‘n’.

Activation at the *aer* promoter requires AR1 of CRP that likely contacts αCTD (Hollands *et al.*, 2007). Our results show that the two αCTDs of Eσ^28^ contact DNA both upstream and downstream of CRP, although note that we cannot prove that both contacts occur simultaneously. The finding that one αCTD binds downstream of CRP at the *aer* promoter was surprising. Structural modelling of the CRP-RNA polymerase-DNA complex at a Class I promoter, where the DNA site for CRP is centred at position −61.5, indicates that one αCTD is tightly sandwiched between CRP and σ^70^, such that it can simultaneously contact DNA, AR1 on CRP and σ^70^ domain 4 (Chen *et al.*, 2003; Lawson *et al.*, 2004). As the DNA site for CRP at the *aer* promoter is located 12 bp downstream, it appears that there cannot be sufficient space for αCTD to fit between the CRP dimer and the promoter-bound sigma factor. We modelled the structure of the CRP-RNA polymerase-DNA complex at the *aer* promoter by combining the crystal structure of the CRP-αCTD-DNA complex (Benoff *et al.*, 2002) with the Eσ^A.^-fork junction DNA structure (Murakami *et al.*, 2002) and, as expected, we found that there is a clash between the predicted locations of the αCTD downstream of CRP and domain 4 of σ, which contacts the promoter −35 element (K. Hollands and D.J. Lee, unpublished). This leads us to propose a model in which the organization of the CRP-Eσ^28^-DNA complex at the *aer* promoter differs from that of the CRP-Eσ^70^-DNA complex at a Class I σ^70^-dependent promoter (Fig. 8A). Our FeBABE footprinting data indicate that the juxtaposition of CRP and the downstream αCTD at the *aer* promoter is the same as at the Class I promoter. This implies that it must be domain 4 of σ^28^ that is positioned differently within the CRP-Eσ^28^-DNA complex, compared with domain 4 of σ^70^ within the CRP-Eσ^70^-DNA complex at a Class I promoter. This is supported by the observation that the −10 and −35 elements at σ^28^-dependent promoters are located 2–3 bp closer together than at promoters served by Eσ^70^ (Fig. 8B), suggesting that the contact site for σ^28^ domain 4 on promoter DNA may lie several bases downstream of that for σ^70^ domain 4 at a σ^70^-dependent promoter. The model, illustrated in Fig. 8A, suggests that domain 4 of σ^28^ is offset by rotation and translocation around the surface of the promoter DNA. It is quite possible that this is its ‘normal’ position at σ^28^-dependent promoters, but this will require further experimental evidence.

Although *E. coli* contains hundreds of transcription activators, there are few examples of factor-dependent activation of promoters recognized by alternative σ factors such as σ^28^. Transcription from promoters served by alternative σ factors is mostly regulated by controlling the expression and activity of the σ factor itself, and by the very stringent promoter recognition properties of the alternative σ factors. It is generally accepted that control over most flagellar genes is exerted by regulating the expression and activity of FlhDC, σ^28^, and the anti-σ factor, FlgM (Chilcott and Hughes, 2000; Keseler *et al.*, 2009). Our findings show that transcription activators can also play an important role in controlling transcription by Eσ^28^.

The similar organization of the *aer* and *trg* promoters suggests a common mechanism of direct activation by CRP. However, we found no evidence for direct regulation by CRP at the six other well-characterized σ^28^-dependent promoters in *E. coli* K-12. This raises the question of why CRP should directly regulate transcription of *aer* and *trg*, particularly when CRP indirectly regulates transcription of all genes in the flagellar cascade by controlling expression of the master regulator, FlhDC. Expression of FlhDC is tightly regulated by multiple transcription factors, including CRP, H-NS and OmpR, and hence the genes of the flagellar cascade are regulated in response to many different environmental inputs (Shin and Park, 1995; Soutourina *et al.*, 1999). It is possible that expression of *aer* and *trg* is required only in response to conditions that induce CRP activity and not in response to other signals that induce the flagellar cascade. Alternatively, expression of *aer* and *trg* may need to be upregulated to a greater extent than other genes when CRP activity is induced. This may be particularly important when levels of Eσ^28^ are low. In these conditions, recruitment of Eσ^28^ by CRP may ensure that the *aer* and *trg* transcription units are preferentially expressed compared with other σ^28^-dependent genes. Note that *aer* and *trg* encode homologous methyl-accepting chemotaxis regulator-type proteins, which interact with the flagellar motor via the CheA/CheY signalling pathway to control the direction of bacterial movement in response to different chemical or energetic signals (Taylor *et al.*, 1999). The direct involvement of CRP in their regulation may therefore simply be a reflection of the role of CRP in facilitating the acquisition and metabolism of nutrients other than glucose (Kolb *et al.*, 1993).

## Experimental procedures

### Strains, plasmids and promoter constructs

The *E. coli* K-12 strains, plasmids and promoter fragments used in this study are listed in Table 2. Standard recombinant DNA techniques were used throughout and all the oligonucleotide primers used are listed in Table S1.

**Table 2:** Strains and plasmids and promoter fragments.

Name	Description	Reference
*E. coli* K-12 strains		
MG1655	F^–^λ^–^*ilvG rfb-50 rph-1*	Blattner *et al.* (1997)
JW1907–1	*fliA::kan rrnB3*Δ*lacZ4787 hsdR514*Δ(*araBAD*)*567*Δ(*rhaBAD*)*568 rph-1*	Baba *et al.* (2006)
M182	*lacX74 galK galU strA*	Casadaban and Cohen (1980)
M182 Δ*crp*	Δcrp lacX74 galK galU strA	Busby *et al.* (1983)
M182 Δ*fliA*	Δ*fliA* lacX74 galK galU strA	This study
M182 Δ*crp*Δ*fliA*	Δ*fliA Δcrp lacX74 galK galU strA*	This study
BL21(DE3)	F^–^*ompT hsdS*_*B*_*(r*_*B*_^–^*m*_*B*_^–^*) gal dcm λ*(DE3 [*lacI lacUV5-T7 gene 1 ind1 sam7 nin5*])	Studier and Moffat (1986)
Plasmids		
pRW50	Broad-host-range *lacZ* expression vector used for cloning EcoRI-HindIII promoter fragments; contains the RK2 origin of replication and encodes Tc^R^	Lodge *et al.* (1992)
pSR	pBR322 derivative, used for cloning EcoRI-HindIII promoter fragments upstream of the λ*oop* terminator	Kolb *et al.* (1995)
pET21a	Protein overexpression vector	Novagen
pKXH100	pET21a carrying *fliA* gene cloned on an NdeI-XhoI fragment	This study
Promoter fragmentsa		
aer200	168 bp EcoRI-HindIII fragment carrying the *aer* regulatory region	Hollands *et al.* (2007)
aer206	Derivative of aer200 with CGA to GCT changes from positions −11 to −9 in the promoter −10 element	This study
aer213	Derivative of aer200 with a T to C substitution at position −32 in the promoter −35 element	This study
aer214	Derivative of aer200 with an A to T substitution at position −30 in the promoter −35 element	This study
aer224	Derivative of aer200 with a G to A substitution at position −28 in the promoter −35 element	This study
aer212	Derivative of aer200, in which the DNA site for CRP is moved to position −41.5	This study
aer211	Derivative of aer200, in which the DNA site for CRP is moved to position −61.5	This study
aer226	Derivative of aer200, in which the DNA site for CRP is moved to position −44.5	This study
aer227	Derivative of aer200, in which the DNA site for CRP is moved to position −54.5	This study
fliC100	EcoRI-HindIII fragment carrying the regulatory region of the *fliC* operon	This study
flgM100	EcoRI-HindIII fragment carrying the regulatory region of the *flgMN* operon	This study
flgK100	EcoRI-HindIII fragment carrying the regulatory region of the *flgKL* operon	This study
motA100	EcoRI-HindIII fragment carrying the regulatory region of the *motABcheAW* operon	This study
tar100	EcoRI-HindIII fragment carrying the regulatory region of the *tar tap cheRB cheYZflgMN* operon	This study
tsr100	EcoRI-HindIII fragment carrying the regulatory region of the *tsr* operon	This study
trg100	EcoRI-HindIII fragment carrying the regulatory region of the *trg* operon	This study

a The base sequence of each of the promoter fragments is shown in Fig. S2.

The Δ*fliA* derivatives of strains M182 and M182 Δ*crp* were constructed by P1 transduction of a *fliA*::*kan* mutation from strain JW1907-1. The kanamycin resistance marker was subsequently removed by expressing the FLP recombinase from plasmid pCP20, as described by Cherepanov and Wackernagel (1995), and the presence of the deletion was confirmed by colony PCR using primers D56550 and D56551.

Plasmid pKXH100 was constructed by cloning an NdeI-XhoI fragment carrying the *fliA* coding sequence from *E. coli* K-12 strain MG1655, amplified by PCR using primers D57845 and D57846, into plasmid pET21a (Novagen). As a result of leaky expression, genes cloned under the control of the T7 promoter in pET21a are expressed even in strains that do not produce T7 RNA polymerase, including M182 (Wu *et al.*, 2005). This activity is independent of the presence of the inducer IPTG (K. Hollands, unpubl. results).

The DNA sequence of each promoter fragment is shown in Fig. S2. Promoter fragments were amplified by PCR from genomic DNA of *E. coli* K-12 strain MG1655, using primers that introduce flanking EcoRI and HindIII sites (listed in Table S1). For promoter activity assays, EcoRI-HindIII fragments were cloned into the *lac* expression vector, pRW50. To construct templates for *in vitro* transcription assays, and to generate DNA fragments for electromobility shift assays and footprinting, promoter fragments were cloned into plasmid pSR. Derivatives of the aer200 fragment carrying point mutations in the −10 or −35 elements (aer206, aer213, aer214 and aer224) were constructed by megaprimer PCR. In a first-round PCR reaction, a megaprimer was synthesized from pSR/aer200 as a template, using a mutagenic primer carrying the desired mutation, and a flanking primer (either D51598 or D53041: see Table S1). The megaprimer was then used in a second-round PCR with the opposing flanking primer and pSR/aer200 as a template to generate a full-length promoter fragment containing the required mutation, which was then cloned into pRW50. The aer212, aer211, aer226 and aer227 fragments were constructed by inserting or deleting DNA between the DNA site for CRP and the −35 element of the *aer* promoter. First, two PCR products were synthesized using pSR/aer200 as a template: one generated using upstream primer D53041 and a downstream primer carrying the insertion or deletion, and a second generated using the downstream primer D51598 and an upstream primer carrying the insertion or deletion (see Table S1). The two PCR products were then annealed via their 26–32 bp overhangs, and the two strands were extended using DNA polymerase to generate a full-length promoter fragment carrying the insertion or deletion. This product was then amplified by PCR using primers D53041 and D51598 and cloned into pRW50.

### β-Galactosidase assays

β-Galactosidase levels in cells carrying promoter::*lacZ* fusions, cloned in pRW50, were measured using the method of Miller (1972). Cells were grown aerobically at 37°C in LB medium. Activities are shown in Miller units (nmol ONPG hydrolysed min^−1^ mg^−1^ dry cell mass), and are averages from at least three independent experiments.

### Primer extension

Transcript start sites were mapped by primer extension as described in Lloyd *et al.* (2008), using RNA purified from strain M182 carrying the aer200 promoter fragment cloned in pRW50 and 5′ end-labelled primer D49724, which anneals downstream of the HindIII site in pRW50. Primer extension products were analysed on denaturing 6% polyacrylamide gels, calibrated with sequencing reactions, and were visualized using a Fuji phosphor screen and Bio-Rad Molecular Imager FX.

### Protein purification

Purified CRP protein was donated by David Grainger (University of Warwick, UK), and wild type *E. coli* core RNA polymerase was purchased from Epicentre Technologies (Madison, WI). His-tagged RNA polymerase α subunits containing a single cysteine residue at position 302 were prepared and labelled with FeBABE as described by Lee *et al.* (2003). FeBABE-tagged α subunits were incorporated into core RNA polymerase using the reconstitution method of Tang *et al.* (1995). Purified σ^28^ and σ^70^ proteins were prepared from BL21(DE3) cells carrying the overexpression plasmid pKXH100, as described by Grainger *et al.* (2008). Eσ^28^ and Eσ^70^ holoenyzmes were made by mixing wild type or FeBABE-labelled core RNA polymerase with an equimolar amount of σ^28^ or σ^70^, and incubating for 20 min at room temperature.

### *In vitro* transcription assays

Caesium chloride preparations of pSR carrying the aer200 promoter fragment served as a template for multiple-round *in vitro* transcription assays, as described by Savery *et al.* (1998). 20 ng pSR/aer200 was incubated in transcription buffer containing 40 mM Tris pH 7.9, 10 mM MgCl_2_, 1 mM dithiothreitol, 100 mM KCl, 100 μg ml^−1^ bovine serum albumin, 200 μM GTP, 200 μM ATP, 200 μM CTP, 10 μM UTP and 5 μCi [α^32^P]-UTP. Where indicated, CRP was included at 100 nM and cAMP at 0.2 mM. Reactions were started by adding Eσ^28^ or Eσ^70^. RNA products were analysed on a denaturing 5.5% polyacrylamide gel and visualized using a Fuji phosphor screen and Bio-Rad Molecular Imager FX.

### Footprinting and EMSA experiments

KMnO_4_ and FeBABE footprinting experiments were performed on PstI-HindIII fragments prepared from caesium chloride preparations of pSR carrying aer200. Fragments were labelled at the HindIII end with [γ-^32^P]-ATP using polynucleotide kinase. KMnO_4_ footprints were performed following the protocol of Browning *et al.* (2009) and FeBABE footprints were carried out as described by Lee *et al.* (2003). Each reaction contained approximately 3 nM labelled PstI-HindIII DNA fragment in 20 mM HEPES pH 8.0, 5 mM MgCl_2_, 50 mM potassium glutamate, 1 mM DTT and 0.5 mg ml^−1^ BSA. KMnO_4_ footprinting reactions contained 0.2 mM cAMP, 100 nM CRP and 50 nM Eσ^28^ or Eσ^70^, as required. FeBABE footprinting reactions contained 0.2 mM cAMP, 100 nM CRP and 200 nM FeBABE-labelled Eσ^28^. The products of KMnO_4_ and FeBABE footprinting reactions were analysed on denaturing 6% polyacrylamide sequencing gels, calibrated with Maxam-Gilbert ‘G + A’ sequencing reactions.

The EMSA experiments were performed using EcoRI-HindIII fragments prepared from pSR derivatives, and end-labelled using [γ-^32^P] ATP and polynucleotide kinase. EMSA reactions were carried out as described by Lloyd *et al.* (1998) and were analysed on 5% polyacrylamide gels. Footprinting and EMSA gels were visualized using a Fuji phosphor screen, and analysed using a Bio-Rad Molecular Imager FX and Quantity One software (Bio-Rad).

## References

[b1] Baba T, Ara T, Hasegawa M, Takai Y, Okumura Y, Baba M (2006). Construction of *Escherichia coli* K-12 in-frame, single-gene knockout mutants: the Keio collection. Mol Syst Biol.

[b2] Barembruch C, Hengge R (2007). Cellular levels and activity of the flagellar sigma factor FliA of *Escherichia coli* are controlled by FlgM-modulated proteolysis. Mol Microbiol.

[b3] Barrett CL, Herring CD, Reed JL, Palsson BO (2005). The global transcriptional regulatory network for metabolism in *Escherichia coli* exhibits few dominant functional states. Proc Natl Acad Sci USA.

[b4] Benoff B, Yang H, Lawson CL, Parkinson G, Liu J, Blatter E (2002). Structural basis of transcription activation: the CAP-alpha CTD-DNA complex. Science.

[b5] Bibikov SI, Biran R, Rudd KE, Parkinson JS (1997). A signal transducer for aerotaxis in *Escherichia coli*. J Bacteriol.

[b6] Blattner FR, Plunkett G, Bloch CA, Perna NT, Burland V, Riley M (1997). The complete genome sequence of *Escherichia coli* K-12. Science.

[b7] Brown CT, Callan CG (2004). Evolutionary comparisons suggest many novel cAMP response protein binding sites in *Escherichia coli*. Proc Natl Acad Sci USA.

[b8] Browning D, Savery N, Kolb A, Busby SJ, LeBlanc B, Moss T (2009). Assays for transcription factor activity. Methods in Molecular Biology: DNA–Protein Interactions.

[b9] Burgess RR, Travers AA, Dunn JJ, Bautz EK (1969). Factor stimulating transcription by RNA polymerase. Nature.

[b10] Busby S, Ebright RH (1999). Transcription activation by catabolite activator protein (CAP). J Mol Biol.

[b11] Busby S, Kotlarz D, Buc H (1983). Deletion mutagenesis of the *Escherichia coli* galactose operon promoter region. J Mol Biol.

[b12] Casadaban MJ, Cohen SN (1980). Analysis of gene control signals by DNA fusion and cloning in *Escherichia coli*. J Mol Biol.

[b13] Chen H, Tang H, Ebright RH (2003). Functional interaction between RNA polymerase alpha subunit C-terminal domain and sigma70 in UP-element- and activator-dependent transcription. Mol Cell.

[b14] Cherepanov PP, Wackernagel W (1995). Gene disruption in *Escherichia coli*: Tc^R^ and Km^R^ cassettes with the option of Flp-catalysed excision of the antibiotic-resistance determinant. Gene.

[b15] Chilcott GS, Hughes KT (2000). Coupling of flagellar gene expression to flagellar assembly in *Salmonella enterica* serovar Typhimurium and *Escherichia coli*. Microbiol Mol Biol Rev.

[b16] Frye J, Karlinsey JE, Felise HR, Marzolf B, Dowidar N, McClelland M, Hughes KT (2006). Identification of new flagellar genes of *Salmonella enterica* serovar Typhimurium. J Bacteriol.

[b17] Gaston K, Bell A, Kolb A, Buc H, Busby S (1990). Stringent spacing requirements for transcription activation by CRP. Cell.

[b18] Germer J, Becker G, Metzner M, Hengge-Aronis R (2001). Role of activator site position and a distal UP-element half-site for sigma factor selectivity at a CRP/H-NS-activated sigma(S)-dependent promoter in *Escherichia coli*. Mol Microbiol.

[b19] Gosset G, Zhang Z, Nayyar S, Cuevas WA, Saier MH (2004). Transcriptome analysis of CRP-dependent catabolite control of gene expression in *Escherichia coli*. J Bacteriol.

[b20] Gourse RL, Ross W, Gaal T (2000). UPs and downs in bacterial transcription initiation: the role of the alpha subunit of RNA polymerase in promoter recognition. Mol Microbiol.

[b21] Grainger DC, Hurd D, Harrison M, Holdstock J, Busby SJ (2005). Studies of the distribution of *Escherichia coli* cAMP-receptor protein and RNA polymerase along the *E. coli* chromosome. Proc Natl Acad Sci USA.

[b22] Grainger DC, Goldberg MD, Lee DJ, Busby SJW (2008). Selective repression by Fis and H-NS at the *Escherichia coli dps* promoter. Mol Microbiol.

[b23] Gruber TM, Gross CA (2003). Multiple sigma subunits and the partitioning of bacterial transcription space. Annu Rev Micriobiol.

[b24] Hollands K, Busby SJ, Lloyd GS (2007). New targets for the cyclic AMP receptor protein in the *Escherichia coli* K-12 genome. FEMS Microbiol Lett.

[b25] Ishihama A (2000). Functional modulation of *Escherichia coli* RNA polymerase. Annu Rev Micriobiol.

[b26] Keseler IM, Bonavides-Martinez C, Collado-Vides J, Gama-Castro S, Gunsalus RP, Johnson DA (2009). EcoCyc: a comprehensive view of *Escherichia coli* biology. Nucleic Acids Res.

[b27] Kolb A, Busby S, Buc H, Garges S, Adhya S (1993). Transcriptional regulation by cAMP and its receptor protein. Annu Rev Biochem.

[b28] Kolb A, Kotlarz D, Kusano S, Ishihama A (1995). Selectivity of the *Escherichia coli* RNA polymerase E sigma 38 for overlapping promoters and ability to support CRP activation. Nucleic Acids Res.

[b29] Koo BM, Rhodius VA, Campbell EA, Gross CA (2009). Mutational analysis of *Escherichia coli* sigma28 and its target promoters reveals recognition of a composite -10 region, comprised of an ‘extended -10’ motif and a core -10 element. Mol Microbiol.

[b30] Lawson CL, Swigon D, Murakami KS, Darst SA, Berman HM, Ebright RH (2004). Catabolite activator protein: DNA binding and transcription activation. Curr Opin Struct Biol.

[b31] Lee DJ, Busby SJ, Lloyd GS (2003). Exploitation of a chemical nuclease to investigate the location and orientation of the *Escherichia coli* RNA polymerase alpha subunit C-terminal domains at simple promoters that are activated by the cyclic AMP receptor protein. J Biol Chem.

[b32] Lloyd GS, Busby SJ, Savery NJ (1998). Spacing requirements for interactions between the C-terminal domain of the alpha subunit of *Escherichia coli* RNA polymerase and the cAMP receptor protein. Biochem J.

[b33] Lloyd GS, Hollands K, Godfrey RE, Busby SJ (2008). Transcription initiation in the *Escherichia coli* K-12 *malI-malX* intergenic region and the role of the cyclic AMP receptor protein. FEMS Micriobiol Lett.

[b34] Lodge J, Fear J, Busby S, Gunasekaran P, Kamini NR (1992). Broad host range plasmids carrying the *Escherichia coli* lactose and galactose operons. FEMS Microbiol Lett.

[b35] Miller (1972). Experiments in Molecular Genetics..

[b36] Murakami KS, Masuda S, Campbell EA, Muzzin O, Darst SA (2002). Structural basis of transcription initiation: an RNA polymerase holoenzyme-DNA complex. Science.

[b37] Park K, Choi S, Ko M, Park C (2001). Novel sigmaF-dependent genes of *Escherichia coli* found using a specified promoter consensus. FEMS Microbiol Lett.

[b38] Rebbapragada A, Johnson MS, Harding GP, Zuccarelli AJ, Fletcher HM, Zhulin IB, Taylor BL (1997). The Aer protein and the serine chemoreceptor Tsr independently sense intracellular energy levels and transduce oxygen, redox, and energy signals for *Escherichia coli* behaviour. Proc Natl Acad Sci USA.

[b39] Rhodius VA, West DM, Webster CL, Busby SJ, Savery NJ (1997). Transcription activation at class II CRP-dependent promoters: the role of different activating regions. Nucleic Acids Res.

[b40] Robison K, McGuire AM, Church GM (1998). A comprehensive library of DNA-binding site matrices for 55 proteins applied to the complete *Escherichia coli* K-12 genome. J Mol Biol.

[b41] Savery NJ, Lloyd GS, Kainz M, Gaal T, Ross W, Ebright RH (1998). Transcription activation at Class II CRP-dependent promoters: identification of determinants in the C-terminal domain of the RNA polymerase alpha subunit. EMBO J.

[b42] Shin S, Park C (1995). Modulation of flagellar expression in *Escherichia coli* by acetyl phosphate and the osmoregulator OmpR. J Bacteriol.

[b43] Smith T, Hoover T (2009). Deciphering bacterial flagellar gene regulatory networks in the genome era. Adv Appl Microbiol.

[b44] Soutourina O, Kolb A, Krin E, Laurent-Winter C, Rimsky S, Danchin A, Bertin P (1999). Multiple control of flagellum biosynthesis in *Escherichia coli*: role of H-NS protein and the cyclic AMP-catabolite activator protein complex in transcription of the flhDC master operon. J Bacteriol.

[b45] Studier FW, Moffatt BA (1986). Use of bacteriophage T7 RNA polymerase to direct selective high-level expression of cloned genes. J Mol Biol.

[b46] Tan K, Moreno-Hagelsieb G, Collado-Vides J, Stormo GD (2001). A comparative genomics approach to prediction of new members of regulons. Genome Res.

[b47] Tang H, Severinov K, Goldfarb A, Ebright RH (1995). Rapid RNA polymerase genetics: one-day, no-column preparation of reconstituted recombinant *Escherichia coli* RNA polymerase. Proc Natl Acad Sci USA.

[b48] Taylor BL, Zhulin IB, Johnson MS (1999). Aerotaxis and other energy-sensing behavior in bacteria. Annu Rev Microbiol.

[b49] Wozniak CE, Hughes KT (2008). Genetic dissection of the consensus sequence for the class 2 and class 3 flagellar promoters. J Mol Biol.

[b50] Wu T, Malinverni J, Ruiz N, Kim S, Silhavy TJ, Kahne D (2005). Identification of a multicomponent complex required for outer membrane biogenesis in *Escherichia coli*. Cell.

[b51] Yu HH, Di Russo EG, Rounds MA, Tan M (2006). Mutational analysis of the promoter recognized by *Chlamydia* and *Escherichia coli* sigma28 RNA polymerase. J Bacteriol.

[b52] Zhao K, Liu M, Burgess RR (2007). Adaptation in bacterial flagellar and motility systems: from regulon members to ‘foraging’-like behavior in *E. coli*. Nucleic Acids Res.

[b53] Zheng D, Constantinidou C, Hobman JL, Minchin SD (2004). Identification of the CRP regulon using *in vitro* and *in vivo* transcriptional profiling. Nucleic Acids Res.

