# Supplementary material for: Activation of σ28-dependent transcription in Escherichia coli by the cyclic AMP receptor protein requires an unusual promoter organization
Source: Mol Microbiol. 2009 Nov 4;75(5):1098–111. doi: 10.1111/j.1365-2958.2009.06913.x (PMC2859248; doi:10.1111/j.1365-2958.2009.06913.x)
Supplement: Supplementary file 1 [file mmi0075-1098-SD1.pdf]

**Hollands *et al*. Supplementary Figure 1**

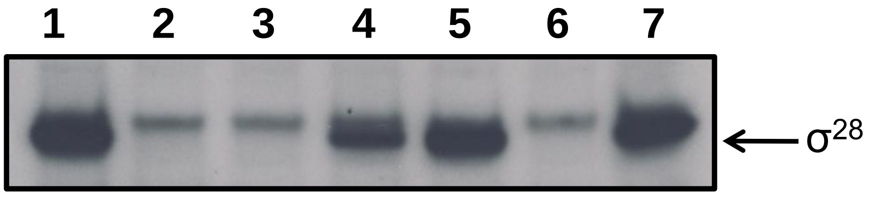


**Figure S1.** Measurement of $\sigma^{28}$ levels in different strains

The figure shows the results of Western blot analysis of $\sigma^{28}$ levels in *E. coli* K-12 strains M182 (lane 1), M182 Δ*crp* (lane 2), M182 Δ*fliA* (lane 3), M182 Δ*fliA* containing pKXH100 ($CRP^{+}$ $FliA^{+}$; lane 4), M182 Δ*fliA*Δ*crp* containing pKXH100 ($CRP^{-}$ $FliA^{+}$; lane 5) or M182 Δ*fliA* containing "empty" pET21a ($CRP^{+}$ $FliA^{-}$; lane 6). Cells were grown to late exponential phase ($OD_{650}$ 0.9-1.1) in LB medium. Cell extracts were electrophoresed on NuPAGE Novex 4-12 % Bis-Tris gels (Invitrogen), transferred to a nitrocellulose membrane and probed with anti-$\sigma^{28}$ primary antibodies (mouse anti-$\sigma^{F}$, Neoclone) and horseradish peroxidase conjugated secondary antibodies (Rabbit anti-mouse IgG HRP-linked antibody, Sigma-Aldrich). The gel was calibrated with 0.5 ng purified $\sigma^{28}$ protein (lane 7). Note that a second non-specific band appears in lanes 1 to 6, above the band corresponding to $\sigma^{28}$.

**Figure S2.**

**aer200**

CRP -35 ***paer*** -10

*GAATTC*ATCAAGGAATTAGATTTGTGTTTAAATCGCAAATTGCGATCTAAATCAAATTAATCGGTTAAAGATAACCGCAGCGGGGCCGACATAAACT**C**TGACAAGAAGTTAACAACCATATAACCT
GCACAGGACGCGAAC**ATG**TCTTCTCATCCGTATGTC*AAGCTT*

**aer206**

CRP -35 ***paer*** -10

*GAATTC*ATCAAGGAATTAGATTTGTGTTTAAATCGCAAATTGCGATCTAAATCAAATTAATCGGTTAAAGATAACCGCAGCGGGGC**GCT**CATAAACT**C**TGACAAGAAGTTAACAACCATATAACCT
GCACAGGACGCGAAC**ATG**TCTTCTCATCCGTATGTC*AAGCTT*

**aer213**

CRP -35 ***paer*** -10

*GAATTC*ATCAAGGAATTAGATTTGTGTTTAAATCGCAAATTGCGATCTAAATCAAATTAATCGGT**C**AAAGATAACCGCAGCGGGGCCGACATAAACT**C**TGACAAGAAGTTAACAACCATATAACCT
GCACAGGACGCGAAC**ATG**TCTTCTCATCCGTATGTC*AAGCTT*

**aer214**

CRP -35 ***paer*** -10

*GAATTC*ATCAAGGAATTAGATTTGTGTTTAAATCGCAAATTGCGATCTAAATCAAATTAATCGGTTA**T**AGATAACCGCAGCGGGGCCGACATAAACT**C**TGACAAGAAGTTAACAACCATATAACCT
GCACAGGACGCGAAC**ATG**TCTTCTCATCCGTATGTC*AAGCTT*

**aer224**

CRP -35 ***paer*** -10

*GAATTC*ATCAAGGAATTAGATTTGTGTTTAAATCGCAAATTGCGATCTAAATCAAATTAATCGGTTAAA**A**ATAACCGCAGCGGGGCCGACATAAACT**C**TGACAAGAAGTTAACAACCATATAACCT
GCACAGGACGCGAAC**ATG**TCTTCTCATCCGTATGTC*AAGCTT*

**aer212**

CRP ▼ -35 ***paer*** -10

*GAATTC*ATCAAGGAATTAGATTTGTGTTTAAATCGCAAATTGCGATCTAAATCAAATTAAAGATAACCGCAGCGGGGCCGACATAAACT**C**TGACAAGAAGTTAACAACCATATAACCTGCACAGGA
CGCGAAC**ATG**TCTTCTCATCCGTATGTC*AAGCTT*

**aer211**

CRP -35 ***paer*** -10

*GAATTC*ATCAAGGAATTAGATTTGTGTTTAAATCGCAAATTGCGATCTAAATCAAATTAATC**CTCGAGGCTAGC**GGTTAAAGATAACCGCAGCGGGGCCGACATAAACT**C**TGACAAGAAGTTAACA
ACCATATAACCTGCACAGGACGCGAAC**ATG**TCTTCTCATCCGTATGTC*AAGCTT*

**aer226**

CRP ▼ -35 ***paer*** -10

*GAATTC*ATCAAGGAATTAGATTTGTGTTTAAATCGCAAATTGCGATCTAAATCAAATTATTAAAGATAACCGCAGCGGGGCCGACATAAACT**C**TGACAAGAAGTTAACAACCATATAACCTGCACA
GGACGCGAAC**ATG**TCTTCTCATCCGTATGTC*AAGCTT*

**aer227**

CRP -35 ***paer*** -10

*GAATTC*ATCAAGGAATTAGATTTGTGTTTAAATCGCAAATTGCGATCTAAATCAAATTAATC**TCGAG**GGTTAAAGATAACCGCAGCGGGGCCGACATAAACT**C**TGACAAGAAGTTAACAACCATAT
AACCTGCACAGGACGCGAAC**ATG**TCTTCTCATCCGTATGTC*AAGCTT*

## fliC100

*GAATTC*TAAGTTTAGCGGTAAACGACGATTGCTGATTTGAAATGGGGGTTAGCGTCGCTTTTTGCGCGGCGGTGAGGCTATCAAGGATGGAACTTAAATCCAGACCTGACCCGACTCCCAGCGATGA

AATACTTGC**CAT**GCGATTTCCTTTTATCTTTCGACACGTAAAACGAATACCGGGGTTATCGGTCTGAATTGCGCAAAGTTTACGTTTAATTGTTTTTTTTAATAGCGGGAATAAGGGGCAGAGAAA

**-10** **p*fliD*** **-35**

AGAGTATTTCGGCGACTAACAAAAAATGGCTGTTTTTGAAAAAAAATTCTAAAGGTTGTTTTACGACAGACGATAACAGGGTTGACGGCGATTGAGCCGACGGGTGGAAACCCAATACGTAATCAAC

**-35** **p*fliC*** **-10**

GACTTGCAATATAGGATAACGAATC**ATG***AAGCTT*

## flgM100

*GAATTC*CAATGTCAAACTGAAACGCGGACGGCTGGATACCCTGCCACCGCGTACGGTGCTGGATATCAATCAACTTGTTGATGCCATTAGCCTGCGCGATCTATCACCCGATCAACCTATCCAGTTA
ACCCAGTTTCGCCAGGCATGGCGGGTAAAAGCGGGACAACGCGTCAATGTGATCGCCAGCGGTGATGGGTTTAGCGCCAACGCAGAAGGTCAGGCGCTGAACAATGCAGCCGTCGCACAGAATGCGC
GGGTGCGCATGGTATCGGGACAGGTAGTCAGCGGCGTTGTTGATGCAGATGGGAATATTCTTATAAACCTGTAAGCTGTTAAAGATTACCCGTCCCTTGCCGATAAATAAGCAACACATGATAAAA
GCGCCCTCAATGAGGAATAAACC**ATG***AAGCTT*

**-35** **p*flgM*** **-10**

## flgK100

*GAATTC*CAAAGCCTCTGGCAACTGGAAAGGGCCAGTTACTGAAATCACCACGACTGAATATGAAAACGGCGAAGCGAAGAAAGTAAAAGCGAAGTTTCGCGTCTACAGCTCGTATCTGGAAGCCTTG
TCGGATTACGTTGGGCTGTTAACGCGTAACCCGCGCTACGCCGCCGTGACGACCGCCGCGAGTGCGGAACAGGGGGCGCAGGCCCTACAGGACGCGGGCTATGCCACCGATCCTCACTATGCCCGCA
AACTCACCAACATGATTCAGCAGATGAAATCGATAAGCGACAAGGTGAGCAAAACCTACAGTATGAACATTGATAATCTGTTCTGAATAACTCAAGTCCGGCGGGTCGCTGCCGATAATACTCTGT
AATTGAAGGCTTATAAGGAACCTCC**ATG***AAGCTT*

**-35** **p*flgK*** **-10**

## motA100

*GAATTC*CCTTGAACAGTGCCCACAAGCAGAAGAAGGACCACTGCTGGCATTAACCCGTGCCTGGACATTGGTGCGGTTTGTTGAAAGTGGATTACTGCAACTTTCCAGCTGCAACTGCTGCGGCGGC
AATTTTATTACCCACGCTCACCAGCCTGTTGGCAGCTTTGCCTGCAGCTTATGTCAACCGCCATCCCGGGCAGTAAAAAGACGTAAACTTTCCCAGAATCCTGCCGATATTATCCCACAACTGCTG

**-35** **p*motA*** **-10**

GATGAACAGAGAGTACAGGCTGTTTAACTGATACGGTGAGGCGCAACATTCCAGCAGCGGTAACGACGTACCGCTGCTTTTTTTTGCCCCAATCGCGCGTTAACGCCTGACGACTGAACATCCTGTC
ATGGTCAACAGTGGAAGGATGATGTC**GTG***AAGCTT*

## tar100

*GAATTC*CGACAACACGGTAGTTATCGTCCTGAATCTCGGACAGCGGGTGGTCGGCATCGTGGTTGACGGCGTCTCAGACGTGCTTTCATTGACGGCGGAGCAAATTCGTCCGGCACCGGAATTTGCC
GTGACGCTTTCAACAGAATATCTCACTGGACTGGGCGCACTGGGCGACCGGATGTTGATTCTGGTGAACATCGAAAAACTGCTGAACAGCGAAGAGATGGCGCTGTTAGATAGCGCGGCGTCAGAAG
TGGCGTAATTCTCCCGATTTCCTCAATTGAAATGAACCCGATGATCTGCGCATCGGGTTTTTTATTTCAATTTCGCGGCGGGTGGCATCAGCAATAAAGTTTCCCCCCTCCTTGCCGATAACGAGA
TCAACTTGTTTTCAGGAAGGTGCCTT**ATG***AAGCTT*

**-35** **p*tar*** **-10**

**tsr100**

*GAATTC*GGGTATGTGCTTGAATATCTTTTTAGTATC**CAT**AGTAAAACCTGGCATGTATTGATTAATAGTTGGCCGAAGCCGTTCTAGGTTTGCGTTGCGTTTGAGGAGGTAAATTGACCGCTAT
CCTGTAGTGATGTTGCCAGAGTTACGCGCGCGTAAAGTAAGGTAAATAACTGAGTGGTTATTTTAGGGATGTAAGCGGTCAGTTTTGCGGTTGAGCGGCAAAGGCGTCGTTTAAGTGACGATAA
ATATGTGATTCATATCACATATTTATATTGTGAATAATTTATGTAAATAATGGCTTTTTAAATTCAGAGTGTGAATAAAATTCACTCGGCGTAATCTCCGCGGGATATTCATAAAGTTTTTCCT
**CRP** **-35**
TTCCAGGCCGAAAATCTTGCATCGGTCCACAGGAAAGAGAAACC**ATG***AAGCTT*
**p*tsr*** **-10**

**trg100**

*GAATTC*AGCCTTTCTCCTTGCCGGATGGCGGGTAAGAGGCTAAGATCTGAATTGCTAGGTTCATTCGTTGGCCTCGGTTGATAGAAATATCGGTCGGGGCCTTCGTCTTTCTGATTCCCGGTTAGCC
TGAAAACAGAAAGTCTCAGGCACCCGCAGGCATCCTATGAGGTTTCCTTAGGGACGAAAATAATCACTTCACGAAATTGCGTGCTGTTTTCCAGAATTTTTCGTCATTCGGGTTAGCCAGTTTAGCC
ATTCGTTACTCTCTTCATTCCAATAGCATTAATTTTCTATGCAATAATTGTTGTAAAAATGTGACGCAAAGAGGTTTTTGGTCATAAGTAATTACCGTCAAGTGCCGATGACTTTCTATCAGGAGT
AAACCTGGACGAGAGACAACGGTA**ATG***AAGCTT* **CRP** **-35** **p*trg*** **-10**

**Figure S2.** DNA sequences of promoter fragments used in this work

The figure shows the sequence of the non-template strand of each EcoRI-HindIII promoter fragment used in this work. In each fragment, the EcoRI and HindIII sites are shown in italics, promoter –10 and –35 elements are boxed, and known or predicted DNA sites for CRP are shown in grey. The translation start site of the gene of interest is shown in bold. Where the translation stop codon of an upstream gene is present on the promoter fragment, this is underlined. Where the translation start site of a divergent gene is present, this is double underlined. In the *aer* promoter fragments, the location of base substitutions in the promoter –10 and –35 elements are highlighted in red, sequence insertions are shown in blue, and the position of deletions are indicated by black triangles above the sequence.

**Table S1.** Oligonucleotide primers used in this work

| Name | Sequence (5’-3’) | Use |
|---|---|---|
| D56550 | CTCATTTCACCCACTAATCG | Upstream primer for screening for presence of Δ*fliA* mutation in strain M182 |
| D56551 | CTTAATGGCCGTCTTTTCAG | Downstream primer for screening for presence of Δ*fliA* mutation in strain M182 |
| D57845 | GGCGCAT ATGAATTCACTCTATACCGCTG | Upstream primer for amplification of the NdeI-XhoI *fliA* fragment for cloning into pET21a |
| D57846 | GCGCCTCGAGTTATAACTTACCCAGTTTAGTG | Downstream primer for amplification of the NdeI-XhoI *fliA* fragment for cloning Into pET21a |
| D49724 | GGTTGGACGCCCGGCATAGTTTTTCAGCAGGTC GTTG | Anneals downstream of the HindIII site in pRW50. Used for mapping transcript start sites using primer extension |
| D53041 | ACGCGGGAATTCATCAAGGAATTAGATTTGTG | Upstream primer for amplification of the aer200 EcoRI-HindIII fragment |
| D51598 | CGCCGCAAGCTTGACATACGGATGAGAAG | Downstream primer for amplification of the aer200 EcoRI-HindIII fragment |
| D56704 | ACCGCAGCGGGGCGCTCATAAACTCTGACAAG | Used in PCR reaction with D51598 to produce megaprimer for construction of aer206 |
| D66004 | GCTGCGGTTATCTTTGACCGATTAATTTGATTTAG | Used in PCR reaction with D53041 to produce megaprimer for construction of aer213 |
| D66005 | GCTGCGGTTATCTATAACCGATTAATTTGATTTAG | Used in PCR reaction with D53041 to produce megaprimer for construction of aer214 |
| D67413 | GCTGCGGTTATTTTTAACCGATTAATTTGATTTAG | Used in PCR reaction with D53041 to produce megaprimer for construction of aer224 |
| D63341 | GATCTAAATCAAATTAAAGATAACCGCAGCG | Used in PCR reaction with D53041 to generate upstream DNA fragment for construction of aer212 fragment |
| D63342 | CGGTTATCTTTAATTTGATTTAGATCGCAATTTG | Used in PCR reaction with D51598 to generate downstream DNA fragment for construction of aer212 fragment |
| D63339 | CAAATTAATCCTCGAGGCTAGCGGTTAAAGATA ACCGCAGCG | Used in PCR reaction with D53041 to generate upstream DNA fragment for construction of aer211 fragment |
| D63340 | ATCTTTAACCGCTAGCCTCGAGGATTAATTTGA TTTAGATCG | Used in PCR reaction with D51598 to generate downstream DNA fragment for construction of aer211 fragment |

| | | |
|---|---|---|
| D67416 | CTGCGGTTATCTTTAATAATTTGATTTAGATCGC AATTTG | Used in PCR reaction with D53041 to generate upstream DNA fragment for construction of aer226 fragment |
| D67415 | GATCTAAATCAAATTATTAAAGATAACCGCAGCG | Used in PCR reaction with D51598 to generate downstream DNA fragment for construction of aer226 fragment |
| D67418 | GTTATCTTTAACCCTCGAGATTAATTTGATTTAG ATCG | Used in PCR reaction with D53041 to generate upstream DNA fragment for construction of aer227 fragment |
| D67417 | AATCAAATTAATCTCGAGGGTTAAAGATAACCG CAGCG | Used in PCR reaction with D51598 to generate downstream DNA fragment for construction of aer227 fragment |
| D63243 | GCGGAATTCTAAGTTTAGCGGTAAACGAC | Upstream primer for amplification of fliC100 EcoRI-HindIII promoter fragment |
| D63244 | GCGAAGCTTCATGATTCGTTATCCTATATTG | Downstream primer for amplification of fliC100 EcoRI-HindIII promoter fragment |
| D63245 | GCGGAATTCCCAATGTCAAACTGAAACGCG | Upstream primer for amplification of flgM100 EcoRI-HindIII promoter fragment |
| D63246 | GCCAAGCTTCATGGTTTATTCCTCATTGAGG | Downstream primer for amplification of flgM100 EcoRI-HindIII promoter fragment |
| D63247 | GCCGAATTCCAAAGCCTCTGGCAACTGG | Upstream primer for amplification of flgK100 EcoRI-HindIII promoter fragment |
| D63248 | GGCAAGCTTCATGGAGGTTCCTTATAAGC | Downstream primer for amplification of flgK100 EcoRI-HindIII promoter fragment |
| D63249 | GGCGAATTCCCTTGAACAGTGCCCACAAG | Upstream primer for amplification of motA100 EcoRI-HindIII promoter fragment |
| D63250 | GCGAAGCTTCACGACATCATCCTTCCACTG | Downstream primer for amplification of motA100 EcoRI-HindIII promoter fragment |
| D63251 | GCGGAATTCCGACAACACGGTAGTTATCG | Upstream primer for amplification of tar100 EcoRI-HindIII promoter fragment |
| D63252 | GGCAAGCTTCATAAGGCACCTTCCTGAAAAC | Downstream primer for amplification of tar100 EcoRI-HindIII promoter fragment |
| D63253 | GCGGAATTCTTCGGGTATGTGCTTGAATATC | Upstream primer for amplification of tsr100 EcoRI-HindIII promoter fragment |
| D63254 | GCCAAGCTTCATGGTTTCTCTTTCCTG | Downstream primer for amplification of tsr100 EcoRI-HindIII promoter fragment |
| D63255 | GCGGAATTCAGCCTTTCTCCTTGCGG | Upstream primer for amplification of trg100 EcoRI-HindIII promoter fragment |
| D63256 | GGCAAGCTTCATTACCGTTGTCTCTCGTC | Downstream primer for amplification of trg100 EcoRI-HindIII promoter fragment |
